# Growing functional modules from a seed protein via integration of protein interaction and gene expression data

**DOI:** 10.1186/1471-2105-8-408

**Published:** 2007-10-23

**Authors:** Ioannis A Maraziotis, Konstantina Dimitrakopoulou, Anastasios Bezerianos

**Affiliations:** 1 Department of Medical Physics, School of Medicine, University of Patras, GR26500 Patras, Greece

## Abstract

**Background:**

Nowadays modern biology aims at unravelling the strands of complex biological structures such as the protein-protein interaction (PPI) networks. A key concept in the organization of PPI networks is the existence of dense subnetworks (functional modules) in them. In recent approaches clustering algorithms were applied at these networks and the resulting subnetworks were evaluated by estimating the coverage of well-established protein complexes they contained. However, most of these algorithms elaborate on an unweighted graph structure which in turn fails to elevate those interactions that would contribute to the construction of biologically more valid and coherent functional modules.

**Results:**

In the current study, we present a method that corroborates the integration of protein interaction and microarray data via the discovery of biologically valid functional modules. Initially the gene expression information is overlaid as weights onto the PPI network and the enriched PPI graph allows us to exploit its topological aspects, while simultaneously highlights enhanced functional association in specific pairs of proteins. Then we present an algorithm that unveils the functional modules of the weighted graph by expanding a kernel protein set, which originates from a given 'seed' protein used as starting-point.

**Conclusion:**

The integrated data and the concept of our approach provide reliable functional modules. We give proofs based on yeast data that our method manages to give accurate results in terms both of structural coherency, as well as functional consistency.

## Background

In the post genomic era one of the most challenging tasks is to reveal modular structures in biological networks, in order to comprehend the function and the dynamics of a living cell [1,2]. The vast amount of genes and proteins that participate in biological networks imposes the need for determination of functional modules within the network in order to reduce the complexity, while these modules will be the first step in deciphering the composite genetic or cellular interactions of the overall network.

The functional module is defined [1] as a group of genes or their products, whose function is separable from those of other modules. The members of the group share genetic or cellular interactions e.g. co-expression, members of the same protein complex, or of the same metabolic or signalling pathway, or of the same cellular aggregate. A very important property of the module is that its members share more interactions among themselves than with the members of other modules, which is obvious in the network topology [1].

The determination of small-scale functional modules, in the form of protein complexes [3] included in them, from large-scale interaction networks is therefore crucial in understanding the relation between the function and organization of a network. Towards this goal, several algorithms, ranging from hierarchical clustering [4] to methods considering local topology-based concepts [5] and graph alignment for determining probabilistic motifs [6], have recently been applied for detecting modules in protein interaction networks. In these approaches the protein network is considered as an unweighted graph, where nodes correspond to proteins and edges to interactions among them. Algorithms based solely on topological aspects manage to capture several features of complex networks. Nevertheless they might prove to be insufficient when applied at protein interaction networks that include special characteristics like inter-module crosstalk.

In this direction recent studies perform clustering after transforming the graph to its weighted correspondent. Various methods have been applied for weighting a graph. Pereira et al. [7] weighted an interaction based on the number of experiments that support it. Rives and Galitski [4] and Arnau et al. [8] weighted the distance between two proteins with the minimum shortest path between them. Even though these approaches assign some kind of confidence score in a certain interaction, still they fail to allocate a possible functional association in a pair of proteins.

The development of high throughput techniques, such as yeast two-hybrid system [9], protein complex identification by mass spectrometry [10,11] and microarray gene expression profiles [12,13], have generated a vast amount of data concerning gene/protein function but the challenging task is to integrate different data sources, in order to find more reliable functional modules in the network topology. Clustering in gene expression, showed that similarity in biological role often corresponds to expression similarity [14] but there are cases where functionally related genes show dissimilar expression profiles or are inversely co-regulated [15].

On the other side protein-protein interaction data reflects the collaboration of proteins to achieve a common goal. Several initial studies [16,17] attempted to investigate possible relation among mRNA and protein expression level. These works resulted in the fact that expression levels and protein abundance are correlated to some degree. These first concepts were furthered investigated by works like Jansen et al. [18] and Tornow and Mewes [19] that revealed a relation between PPI and gene expression, by associating both data sources. Specifically it was shown that the subunits of a permanent complex are co-expressed whereas protein interactions that descend from transient complexes or from yeast two-hybrid experiments have weak relationship with gene expression. In addition, recent studies concentrated on inferring gene function based on both data sources [20-22].

In our study we introduce a new method for integrating gene expression data and protein-protein interactions (PPI) in order to determine functional modules. Specifically, given a graph describing a set of proteins and the experimentally determined interactions among them, we assign a non-negative weight to each one of these interactions. This weight descends from clustering the gene expression profiles of the corresponding proteins, an approach that to the best of our knowledge has not been used before in the literature. Our goal is to discover biologically relevant PPI subnetworks, out of a larger network, whose proteins interact significantly. Therefore, after the creation of the weighted graph we suggest a new algorithm for the determination of functional modules within the original PPI graph starting from a kernel protein group that originates from a 'seed' protein.

We prove based on data of *Saccharomyces cerevisiae *that the integrated approach we present manages to adequately and solely identify coherent modules and at the same time outperforms other methods in the literature.

## Results and discussion

In our approach, we have reinforced the simple graph structure by integrating gene expression profiles and protein interactions of *Saccharomyces cerevisiae*, in order to detect valid functional modules, in terms of protein complexes they contain. We created a weighted PPI graph, in which the weight of the interactions originates from clustering the gene expression profiles of the corresponding proteins. Specifically the weight of a PPI derives from a metric that takes into account the distance of the corresponding gene expression profiles from the centroids of their clusters, as well as the distance between the two cluster centroids themselves. Next we applied at the enriched PPI graph a new algorithm called DMSP (Detect Module from Seed Protein), whose role was to construct functional modules starting from a 'seed' protein that belongs to our dataset. All the steps of this procedure are described in detail at the Methods section.

The derived modules are evaluated in terms of functional enrichment in GO terms, structural coherency and coverage in protein complexes. Additionally we compare the modules of our integrated method with the modules descending from PPI data only (named hereafter PPI method), after the application of the algorithm described in the study of Wu and Hu [23] (named hereafter W&H algorithm) to our PPI dataset.

### Case study evaluation

To our knowledge there are no studies similar to ours, except one presented in [23], whose algorithm builds likewise modules from seed proteins based however only on PPI data. Of course in literature there many available algorithms for partitioning the PPI graph [24,25], nevertheless they have different theoretical concept and implement an overall clustering of the graph, whereas DMSP as well as other similar methods like the W&H algorithm focus on certain parts of the PPI network. Specifically these algorithms concentrate on specific fractions of the graph structure in the sense that the determination of the functional modules is directed by the initial protein seed given as input to the algorithm. Thus, in order to avoid the probable false superiority of our algorithm against an overall clustering approach, we will compare the performance of our integrated method against a method restricted to protein-protein interaction data only [23].

**Figure 1:**
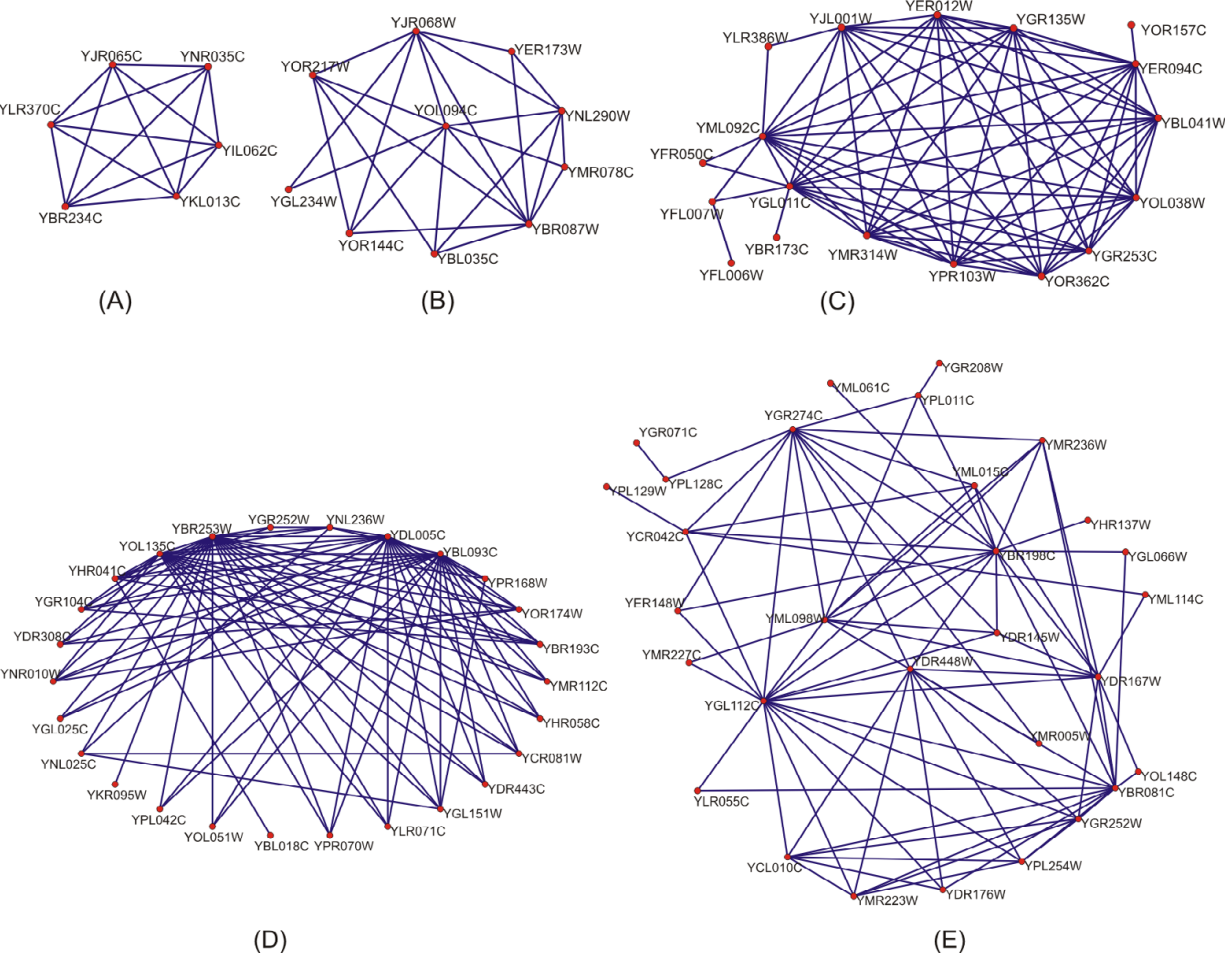
**Examples of modules with various sizes resulting from our integrated method**. Functional modules determined by DMSP are represented as graphs, where vertices represent proteins and edges represent interactions that have been experimentally determined. In this figure, we give some examples of modules with less than 20 members (A, B, C) and modules with more than 20 members (D, E). In each one of these modules protein complexes were identified. This module **(A) **contains the Arp2p/Arp3p complex **(B) **the Replication Factor C complex **(C) **the 20S Proteasome that was discovered in its entirety, **(D) **the SRB-Srb10p complexes and **(E) **the ADA-SAGA-TFIID complexes.

In this work we constructed several functional modules starting from a randomly selected 'seed' protein. This protein is part of a known protein complex that we want to examine. Our approach manages to prevail over the method of Wu and Hu [23] in all the cases examined in this work. An example is the module of the SRB-Srb10p complexes (Figure 1D) that was built with SRB6 as seed protein. Srb10p is a cyclin dependent kinase complex (4 members) that phosphorylates RNA polymerase II and participates in transcription [26]. SRB is a mediator complex that transmits regulatory signals from DNA-binding transcription factors to RNA polymerase II [27]. Our method succeeded to find 4/4 proteins of Srb10p complex and 18/21 proteins of SRB complex, in a module of 26 members (Table 1). In comparison to the similar study [23], which found 14/21 proteins of SRB in a module of 39 members along with other complexes, our results are better and the method captured the Srb10p complex that is associated to SRB in transcriptional level.

**Table 1:** Statistical/Functional data of determined modules.

**Functional Modules**				**Protein Complexes in Functional Modules**	
**Seed Protein**	**Connectivity Density**	**Members**	**R**	**MIPS Protein Complex**	**p-Value**

YGR210c	0.61	12	0.91	1. eIF2B* 2. eIF2* 3. Cytoplasmic translation init	<1e-14 5.4e-10 <1e-14
CDC39 (YCR093w)	0.73	20	0.75	1. NOT complex* 2. DNA polymerase alpha (I) – primase* 3. CCR4 complex	1.9e-13 7.5e-11 <1e-14
TAF5 (YBR198c)	0.79	30	0.83	1. TFIID* 2. TAFIIs* 3. SAGA* 4. ADA	<1e-14 <1e-14 <1e-14 <1e-14
RPB5 (YBR154c)	0.73	32	0.77	1. RNA polymerase III 2. RNA polymerase I	<1e-14 <1e-14
SRB6 (YBR253w)	0.77	26	0.89	1. RNA polymerase II holoenzyme 2. Kornberg's mediator (SRB) 3. Srb10p* complex	<1e-14 <1e-14 2.3e-10
PRE3 (YJL001w)	0.83	18	0.81	1. 20S proteasome* 2. 26S proteasome	<1e-14 <1e-14
SEC10 (YLR166c)	0.75	10	0.80	1. Exocyst complex	<1e-14
RFC5 (YBR087w)	0.84	10	0.92	1. Replication factor C complex*	<1e-14
ARC18 (YLR370c)	0.77	6	0.87	1. Arp2p/Arp3p complex	<1e-14
SEC27 (YGL137w)	0.97	10	1.0	1. TRAPP	<1e-14

The sub-columns of 'Functional Modules' contain statistical data for various modules of different size, while in the two last sub-columns we display the MIPS description of the basic complexes of each module and their corresponding p-values. (*) DMSP manages to find the entire complex.

A second example is the module of DNA polymerase alpha (I) primase-NOT-CCR4 complexes, identified with CDC39 as seed protein (Table 1). The DNA polymerase alpha (I) – primase complex contains 4 proteins, all involved in DNA replication [28]. Its structural and biochemical properties are conserved across a wide range of species. NOT complex contains 5 proteins and CCR4 complex contains 13 proteins. Both complexes are gene expression regulators and participate in transcription and in DNA damage response. Our method succeeded to find 5/5 proteins of NOT complex, 4/4 proteins of DNA polymerase alpha (I) – primase complex and 7/13 proteins of CCR4 complex in a module of 20 members. Again in comparison to the study of Wu and Hu [23] our method gave better results since the module they determined had 40 members. Another interesting module is the Arp2p/Arp3p complex (Figure 1A) that was constructed with ARC18 as 'seed' protein. This complex involves 6 proteins, which take part in actin-filament organization and influence the maintenance of actin-based structures [29]. Our method succeeded to find 5/6 proteins of this complex in a module of 6 members (Table 1). Again in comparison to the associated study [23], our method gave better results, since their module consisted of 20 members.

Finally, we present the module of ADA-SAGA-TFIID complexes (Figure 1E) that was built with TAF5 as seed protein. ADA complex is part of a larger complex named ADA complexes, which contains proteins that play an essential role in organization of chromosome structure [30] and in transcription. SAGA complex is a multifunctional co-activator that regulates transcription by RNA polymerase II [31]. TFIID contains proteins mostly involved in initiation of RNA polymerase II transcription and in transcriptional control [32]. SAGA and TFIID are closely related since both participate in the expression of RNA polymerase II transcribed genes [31]. Our method succeeded to find over 95% of the ADA and SAGA complexes, and 13/13 of TFIID complex in a module of 30 members (Table 1). In comparison to the study of Wu and Hu [23], our method again presents better results since they identified in a module of 39 members, parts of the SAGA and TFIID complexes.

The defect of the algorithm used in the cross-checked study, is that it tends to build large modules in order to capture certain complexes, whereas DMSP builds small-sized modules where almost all members are part of a complex. In this way the condition proposed by Spirin and Mirny [33], that the size of a module should range within 5–25 members, is satisfied.

Nevertheless, if we want to examine thoroughly our approach, we have to extend the conducted experiments beyond isolated and individual examples. In a task like this the eloquent question is how we choose the 'seed' proteins. The answer lies in the concept of protein complexes that have the leading role in the overall evaluation of our method. Specifically we chose to examine complexes with more than 5 members and 80% coverage in terms of proteins included in our data set. The first limitation is based on the fact that it is very probable for small protein complexes to be contained in large functional modules by chance. The second limitation deals with the way we gathered data for this study (i.e. we decreased the number of PPI, preserving only the highly confident ones). So there are cases where the method fails to give good results for certain complexes. In other words while the information we have included in the study is adequate, for certain cases can be insufficient. Most of the studies, that exploit PPI data, avoid weeding out the false positive and false negative interactions, thus this 'burden' can result in misleading functional modules. However, the limitations of our dataset offer the advantage of predicting highly confident functional modules leaving though some space for interactions that are not yet established but have high probability to be correct. One example displaying this characteristic is the module of H+-transporting ATPase vacuolar that was built from VMA8 as seed protein. The role of V-ATPase complex in eukaryotic cells (15 members) is to couple the energy of ATP hydrolysis into proton transport across intracellular and plasma membranes [34]. Our PPI network contains 9/15 of the members of this complex and the rest 6 proteins are not included. This case shows that our information is insufficient in this case. Our method succeeded to capture 8/9 proteins of this complex in a module of 10 members. In order to find 9/9 members the module must have 29 members. This abrupt enlargement of the subnetwork results from the 6 proteins that we lack, but at the same time underlines the efficiency of the proposed integrated method.

We return now to the issue of deciding a set of proteins that will be used as 'seeds' for the construction of functional modules throughout the whole data set, in order to evaluate better our method. Towards this goal we have followed a methodology where we sorted the members of a certain complex, that fulfils the two limitation discussed above (i.e. more than 5 members and more than 80% in data coverage). Specifically we sorted its members starting with the one that had the largest number of neighbours (independently of the module) down to the member with the smallest number of neighbours. Subsequently we created a set with the top 20% of the sorted members. These protein sets were used as 'seed' proteins in our method. Our results show that a large amount of members in the initially selected protein set can be used as 'seed' resulting always in the same module. An example is the module of 20S proteasome complex (Figure 1C) which is identified using one of the SCL1, PRE1, PRE3, PRE9, PRE10, PRE2 proteins as seed. This complex (15 proteins) is involved in protein degradation through the ubiquitin/proteasomal pathway [35]. Our method found 15/15 proteins of this complex in a module of 18 proteins (Table 1). This remark elucidates the flexibility of our algorithm that manages to capture the 'correct' modules independently of the 'seed' protein. In other words the vital need is not the determination of only one protein but of a candidate population of proteins that all will end up in the same result. Once the candidate population is determined the final choice of the 'seed' protein can be random.

### Validation of functional modules

In order to test the validity of the functional modules determined by our integrated approach we will employ three criteria. The first one includes the Gene Ontology annotation scheme to gain insights in the common underlying biological processes of the modules. The second one utilizes the metric of connectivity density to show how well connected the determined modules are, while the last one uses a metric we call complex coverage for checking their biological relevance. At this point it is worth mentioning that in order to show that our integrated approach is superior to a method restricted to PPI data only, we applied the W&H to our PPI dataset and examined the resulted modules with all 3 validation criteria mentioned above. However the forthcoming comparisons have different interpretation from the case study evaluation where we cross-checked our modules with the modules of the associated study as mentioned in the literature.

Additionally and in order to have a more coherent evaluation we compared the results of our approach in terms of functional enrichment analysis with a method using gene expression data only. Specifically following the methodology described in [36,37] we have created a co-expression network, where every gene expression profile corresponds to a node. Under this scheme a co-expression network is based on the absolute value of correlation coefficient among genes and is represented by an adjacency matrix. After creating the network we applied an average linked hierarchical clustering algorithm using *diss*(*i*, *j*) = 1 - *a*_*ij *_= 1-|*cor *(*x*_*i*_, *x*_*j*_)|^6^, as a dissimilarity measure where *x*_*i *_represents the expression profile of the *i*-th gene.

**Figure 2:**
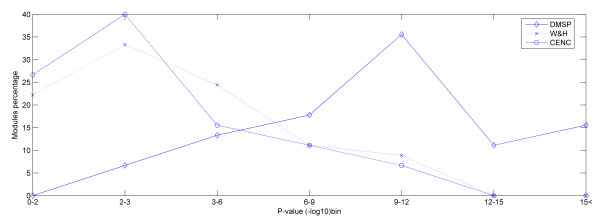
In this diagram we present the functional enrichment of modules in biological process GO terms. It is evident that the majority (75%) of the modules extracted by DMSP has p-value bins larger than 9, whereas 80% of the modules resulting from the PPI method (W&H) and 83% of the modules determined by clustering the co-expression network (CENC) have p-value bins ranging between 0 to 6.

Returning to the issue concerning the validation check of the DMSP extracted functional modules, we firstly substantiated the biological significance of our modules by estimating the GO biological process term co-occurrence with the use of the SGD GO Term Finder [38]. This tool calculates a p-value that represents the probability of observing the co-occurrence of certain proteins with a specific GO annotation in a module by chance based on binomial distribution. The statistical significance of a module in a GO term is increased as the p-value gets lower. We scanned all modules resulted by DMSP as well as the modules descended from the W&H algorithm in order to examine their functional enrichment in biological process GO terms. In Figure 2 it is obvious that the majority of DMSP modules (75%) has p-value bins larger than 9, whereas the p-value bins of 80% of the W&H modules and 83% of the modules determined by clustering the co-expression network range between 0 and 6. This comparison establishes the superiority of our integrated method versus approaches using only one of the two kinds of genomic data, since the objective of a module-detecting study is to identify modules whose members participate up to a great degree in the same biological processes and thus fulfill the biological interpretation of the term 'functional module'. Also further in our validation process we examined the modules through the protein complexes they assess. Protein complexes are too strong indicatives that the members they contain take part in the same biological process, since the definition of protein complex requires its members to accomplish a distinct task at the same time and place [33]. Specifically one module detected by our integrated approach is characterized by APC (Anaphase Promoting Complex), an ubiquitin-protein ligase, which is responsible for the degradation of mitotic cyclins. Specifically it destroys anaphase inhibitory proteins and triggers the separation of sister chromatids [39]. In this way it creates the low CDK state essential for cytokinesis and for reforming the pre-RCs complexes needed for another round of genome replication. Our algorithm managed to include all its 11 proteins in a module of 11 members. The GO Term Finder showed that this module is statistically enriched in two GO terms, cyclin catabolic process (GO: 0008054) and mitotic spindle elongation (GO: 0000022), with the p-value < e-14 in both cases.

The verification of how well connected are the members of a module will be resolved by employing the connectivity density. Connectivity density is the ratio of the total in-module degrees of the vertices to the total number of their connections. This metric has been used in many other similar studies [3]. The connectivity density has a value between 0 and 1, but its value should fluctuate between 0.5 and 1 if we want to have well established results. The higher the value of connectivity density the more probable the determination of a functional module is correct.

**Figure 3:**
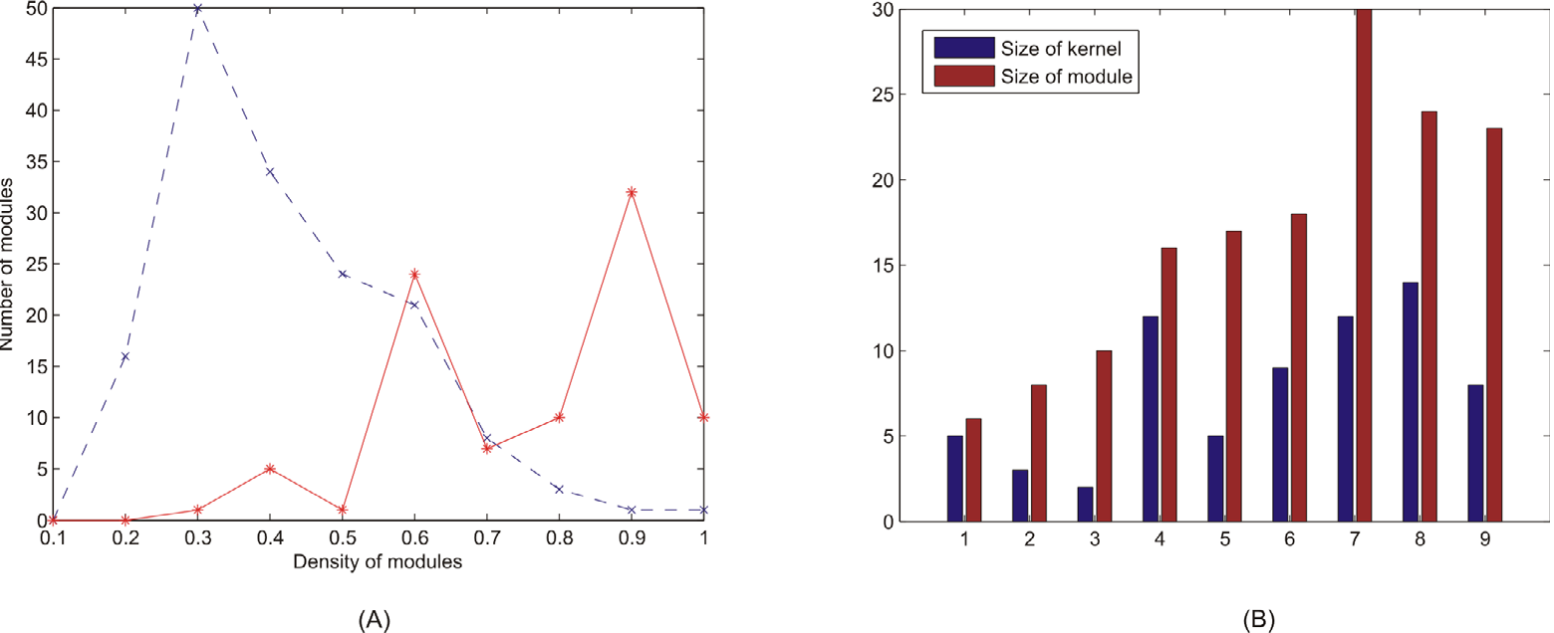
In **(A) **we present the number of modules determined by DMSP on various values of connectivity density. In blue dotted line we depict the number of functional modules, by keeping looser criteria for the determination of the modules. When we apply stricter criteria (as in red dotted line) there is a slight decrease in the number of the modules but at the same time the connectivity density values are better. In **(B) **figure we display the initial size of the kernel, as well as the final size of the module, for modules with various sizes, and densities above 0.5. As we can see, DMSP manages to expand the initial size of the kernel in various degrees in order to determine the most coherent module each time.

Based on the concept of connectivity density we tested the capabilities of our algorithm by tuning the parameters from the zenith (strictest) up to their nadir (loosest) and we checked how connectivity density varies accordingly. As we see in Figure 3A the selection of looser criteria (blue dotted line) leads to an increased number of modules but the densities range from 0.3 to 0.5. On the other hand when we apply gradually stricter parameters (red dotted line) connectivity density also increases gradually. It is obvious though that we assess a smaller number of modules but the density of the majority of those ranges from 0.6 to 0.9. We have already mentioned that it is essential for the modules to have density above 0.5, so the strictest parameters were the chosen ones in our study.

Another worth mentioning aspect of our integrated method is the adaptability of our algorithm concerning the size of the module versus the size of the kernel protein set. In Figure 3B we display various cases dealing with the size of the kernel and of the module. It is obvious that the size of the module rises not analogically with the size of the kernel but rather in an 'adaptive' manner. This property of the DMSP algorithm pinpoints its ability to expand the kernel independently of its size and this procedure continues until the algorithm can no more add proteins to the expanding kernel. This property is demonstrated through Replication factor C complex (Figure 1B). This complex (5 members) functions as a DNA-dependent ATPase and takes part in DNA replication [40] and DNA repair [41]. Our method succeeded to find all 5 proteins in a module of 10 proteins (Table 1), with the size of initial kernel equal to 6 members. In comparison to the study of Wu and Hu [23] our results are better, since their study identified all 5 members in a module of 17 members. Our method managed to control the growing procedure to such a number of proteins in the final module which is very close to the size of the kernel.

The extracted functional modules were evaluated from the biological point of view by a metric that takes into consideration the number of protein complexes that have been successfully encapsulated in a module. Before describing it, we are inserting a new term named basic complex. Basic complex is a complex within a module where the p-value is less than a certain limit which in this work is set to be 10^-10^. P-value is a measure concerning the degree up to which a module manages to capture a particular complex. This metric is mathematically described by:

R(Mi)=1−12(∑t=1L(|Nct|−|NFt|)∑t=1L|Nct|+|Mi|−∑t=1L(|Mi|⊕|NFt|)|Mi|)
 MathType@MTEF@5@5@+=feaafiart1ev1aaatCvAUfKttLearuWrP9MDH5MBPbIqV92AaeXatLxBI9gBaebbnrfifHhDYfgasaacH8akY=wiFfYdH8Gipec8Eeeu0xXdbba9frFj0=OqFfea0dXdd9vqai=hGuQ8kuc9pgc9s8qqaq=dirpe0xb9q8qiLsFr0=vr0=vr0dc8meaabaqaciaacaGaaeqabaqabeGadaaakeaacqWGsbGudaqadaqaaiabd2eannaaBaaaleaacqWGPbqAaeqaaaGccaGLOaGaayzkaaGaeyypa0JaeGymaeJaeyOeI0YaaSaaaeaacqaIXaqmaeaacqaIYaGmaaWaaeWaaeaadaWcaaqaamaaqahabaWaaeWaaeaadaabdaqaaiabd6eaonaaDaaaleaacqWGJbWyaeaacqWG0baDaaaakiaawEa7caGLiWoacqGHsisldaabdaqaaiabd6eaonaaDaaaleaacqWGgbGraeaacqWG0baDaaaakiaawEa7caGLiWoaaiaawIcacaGLPaaaaSqaaiabdsha0jabg2da9iabigdaXaqaaiabdYeambqdcqGHris5aaGcbaWaaabCaeaadaabdaqaaiabd6eaonaaDaaaleaacqWGJbWyaeaacqWG0baDaaaakiaawEa7caGLiWoaaSqaaiabdsha0jabg2da9iabigdaXaqaaiabdYeambqdcqGHris5aaaakiabgUcaRmaalaaabaWaaqWaaeaacqWGnbqtdaWgaaWcbaGaemyAaKgabeaaaOGaay5bSlaawIa7aiabgkHiTmaaqahabaWaaeWaaeaadaabdaqaaiabd2eannaaBaaaleaacqWGPbqAaeqaaaGccaGLhWUaayjcSdGaeyyLIu8aaqWaaeaacqWGobGtdaqhaaWcbaGaemOrayeabaGaemiDaqhaaaGccaGLhWUaayjcSdaacaGLOaGaayzkaaaaleaacqWG0baDcqGH9aqpcqaIXaqmaeaacqWGmbata0GaeyyeIuoaaOqaamaaemaabaGaemyta00aaSbaaSqaaiabdMgaPbqabaaakiaawEa7caGLiWoaaaaacaGLOaGaayzkaaaaaa@82EC@

where M_i _is the members of the i-th functional module, N_C_^t ^is the size of a real basic complex, and N_F_^t ^the size of the corresponding determined complex. L determines the number of basic complexes within the module. Value of R(Mi) ranges from 0 to 1, with 1 being the ideal case where our method manages to find all the proteins of the all basic complexes and 0 as the extreme case where no member of any basic complex has been found (Table 1). In our study we focused on a certain number of modules that we denominate confident and reliable. Thus, after running the algorithm with strict criteria for all selected seed proteins we ended up with 78 modules. However, 45 of those had connectivity density over 0.5 and fulfilled the two constraints discussed above. Subsequently, this set of 45 modules is supported by our integrated method as reliable and accurate.

**Figure 4:**
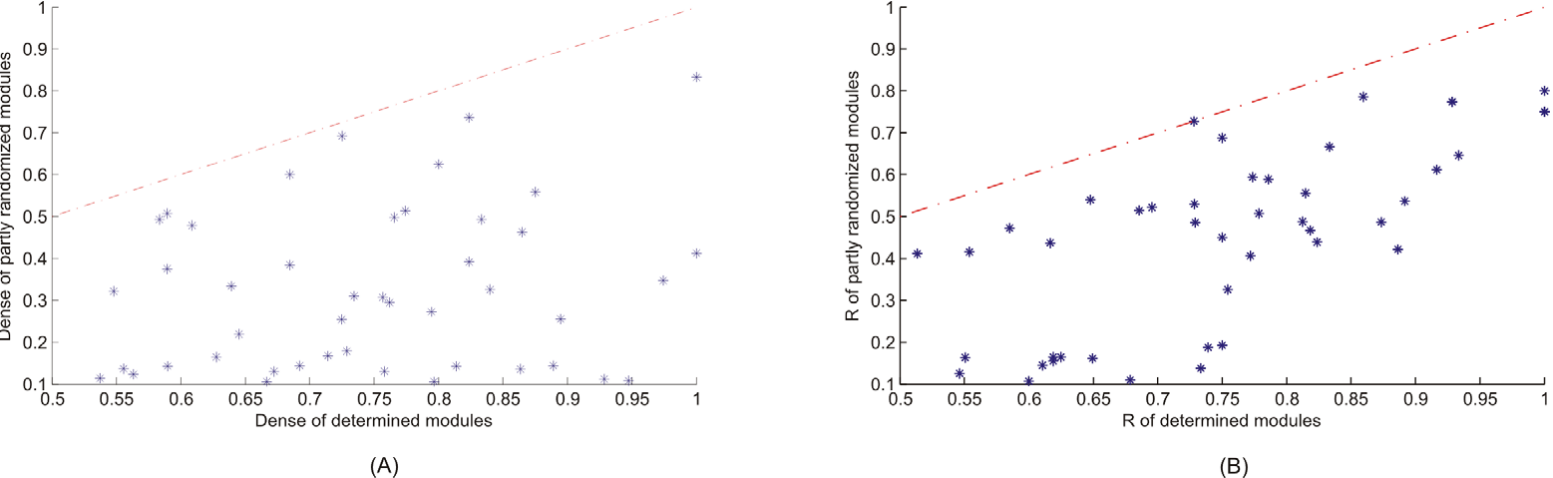
Scatter plots of statistical metrics for the derived and artificial functional modules. Each data point represents statistical value for a certain functional module (x-axis) and its artificially created corresponding module (y-axis). The red dashed line corresponds to the line y = x. When a data point is below the line then the artificial module has a lower statistical value than the derived one, while the opposite stands for the case a data point is above the line. When the data point is on the line it means that the derived and its corresponding artificial module have the same value. **(A) **The metric used in this plot is connectivity density, which is a measure of how densely connected is a specific module. **(B) **Representation of R measuring the coverage in protein complexes of a detected functional module. It is evident from both diagrams that in all cases the derived from DMSP functional modules have better statistical values than the artificial ones.

Next step was to create artificial modules so as to make feasible the comparison, in terms of both connectivity density as well as complex coverage, with the derived modules. Specifically we have implemented a randomization procedure, where we replaced 30% of the proteins of the modules with others that connect to the members of the module but do not belong to it. This randomized replacement was realized iteratively 15 times for each one of the 45 modules and the average connectivity density was measured (Figure 4A). Each one of the artificial modules is the average of these 15 random replacements. Also the protein complex coverage R(Mi) was estimated before and after the randomization (Figure 4B) for all 45 modules following the concept realized in connectivity density. It is evident in Figure 4A that all artificial modules have smaller connectivity density than the derived ones, indicating that our algorithm unveils functional modules with self-reliance. As it is obvious (Figure 4B) the determined modules have always better coverage in protein complexes than the artificial ones. In other words the functional modules identified by our integrated method are dominated by known protein complexes, many of which are sometimes identified in their entirety. This remark elucidates the biggest accomplishment of our study i.e. to unravel from the 'spider-like' protein web subnetworks that have a unique and distinct biological role.

**Figure 5:**
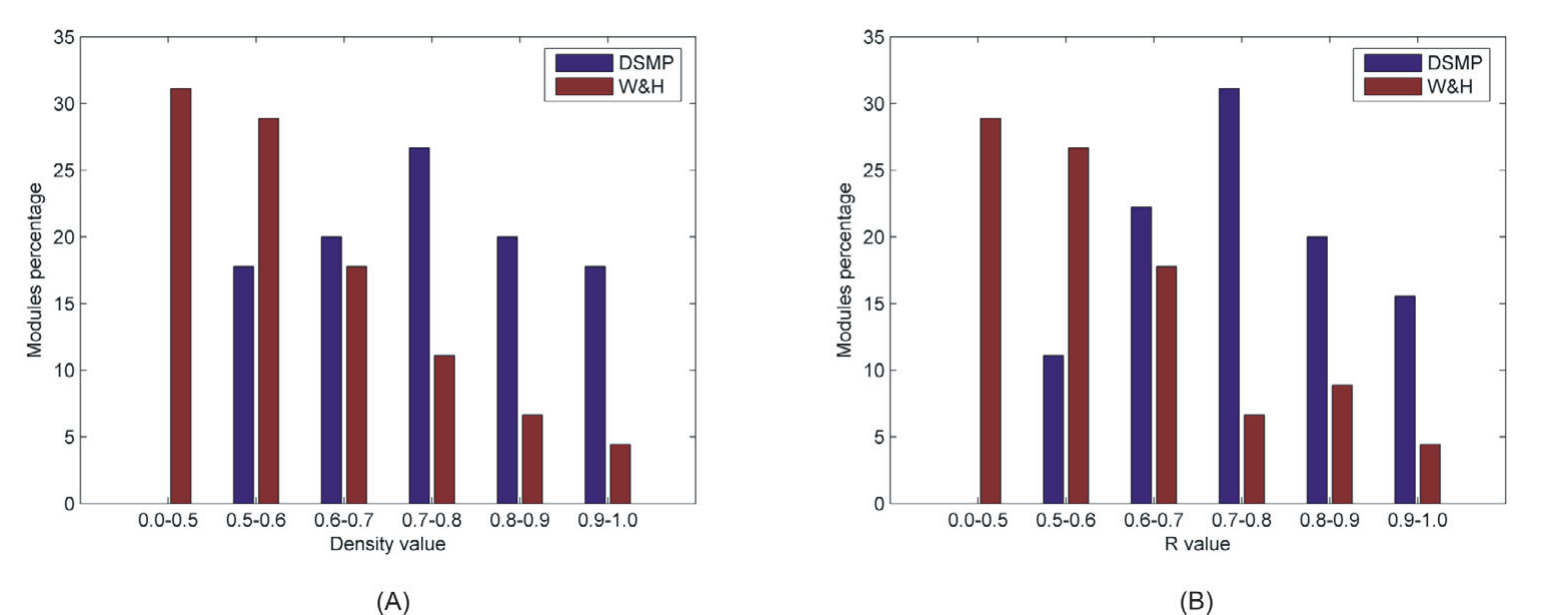
In **(A) **bar plot we compare the modules descending from our integrated method and the PPI method in terms of connectivity density. It is obvious that 75% of the modules descending from the PPI method have connectivity density ranging between 0 and 0.7, whereas the connectivity density of our modules (83%) varies between 0.6 and 1. In **(B) **accordingly we compare the modules in regard to the metric R. It is apparent that 75% of the modules resulting from the PPI method have R fluctuating between 0 and 0.7, whereas the R of our modules (90%) varies between 0.6 and 1.

Lastly in our analysis we compare the modules of our integrated method with the modules of the PPI method by checking their connectivity density as well as the complex coverage (R). In Figure 5A it is apparent that the connectivity density in 75% of the W&H modules ranges between 0 and 0.7, whereas 83% of the DMSP modules have connectivity density varying between 0.6 and 1. This visible difference is explained by the way the two algorithms constitute neighbourhoods around the seed protein. On one hand DMSP algorithm embraces only proteins that are strongly supported both by PPI as well as by gene expression data. In this way the resulting modules are by far more densely connected because the respective proteins were allowed to enter the modules after complying with the constraints raised by both types of data. On the other hand W&H algorithm based only on the topological properties of the PPI network constructs modules whose size is in many cases bigger, since it includes proteins that are not so well connected to the other members, thus the connectivity density decreases enormously. The same scene is repeated when it comes to the metric R. In Figure 5B it is evident that R in 75% of the W&H modules ranges between 0–0.7, whereas 90% of the DMSP modules have R varying between 0.6–1. Again this difference emphasizes the superiority of our integrated method and the reason is easily understood. The metric R, as defined, is affected not only by the number of protein complex members encapsulated in the module but also by the size of the respective module. Based on this the W&H algorithm loses against DMSP because we observed that its modules are in most cases bigger in size, with the majority of the members sharing same functional annotation but with poor protein complex coverage. The two bar plots of Figure 5 as well as the diagram of Figure 2 constitute the proofs that our integrated approach prevails over the PPI method both from biological as well as by topological point of view.

## Conclusion

The post genomic era poses two closely connected challenges. The first challenge is the consolidation of all kinds of high-throughput data, which in fact all describe the same complex dynamics of the living cell but through different perspectives. The second challenge is to unlock the hidden biologically meaningful structures (functional modules) that lie in these integrated constructions. Our method is based on the integration of protein interaction and gene expression profile data, whose association has already been validated by other studies. We overlaid the information descending from gene expression clustering as weights onto a PPI yeast graph, with quite highly confident interactions. This accomplishment fulfils the need for consolidation.

Subsequently, we propose a new algorithm (DMSP), which constitutes subnetworks that originate from a kernel protein set built up from a 'seed' protein. In order for us to characterize these subnetworks as functional modules we checked their biological relevance. This was achieved through some criteria such as the functional enrichment of the resulting modules in GO terms, their connectivity density, their coverage in known complexes and the degree of resemblance between the modules of our integrated approach and the modules of an associated work. Our results show that the majority of our modules have both connectivity density as well as R, over 0.7. Additionally the modules of our integrated method outperform the corresponding modules of the PPI method in all validation criteria. Thus, we succeeded to fulfil the second expectation mentioned above.

It is obvious that the concept of our method can be extended to any set of cellular and genetic data. In future work we plan to use this integrated approach and the concept of DMSP as a basic component for an algorithm that will perform graph clustering in the overall PPI network, again having as goal the identification of functional modules as well as the functional dependencies between modules.

## Methods

The modeling of PPI networks by simple graph structures is common in many applications, including the determination of protein complexes, within protein networks. Protein complexes seem to have a corresponding analog in the graph structure-like of the protein network. This analog corresponds to dense subgraphs of the initial graph, in other words proteins of a specific complex are found to be highly interactive with each other [42]. Following, we will describe in detail the method we propose in order to weight the original PPI graph, as well as provide an extended description of the proposed DMSP algorithm.

### Protein interaction data

A common problem that appears when dealing with protein interaction data, obtained by high-throughput techniques [11,43,44] is confidence. Therefore we used protein interaction data from studies that assigned a confidence score to the protein interaction data [45,46] and downloaded these datasets from their websites. From the first data source we selected protein interaction data with high and medium confidence score (excluding interactions with low score), while from the second source interaction data with likelihood ratio > 1. The final dataset was formed by the combination of the two data sources and after removing redundancy contained 8081 interactions between 2985 proteins.

### Gene expression data

We used yeast expression dataset to evaluate our method. The data contains cell cycle related profiles using cdc15 synchronization over three cell cycles [47]. The expression data is available in the form of a matrix with N rows and D columns. The columns represent the 24 time points and the rows the gene profiles during the cell cycle. The data used in the calculations had already been preprocessed. We chose cell cycle data because it highlights the dynamic character of genes during the phases of the cycle and pinpoints the periodicity of certain genes at certain phases, revealing their cell-cycle regulation.

The initial set of 2985 genes was clustered in 16 groups according to the fuzzy c-means algorithm. It is worth mentioning that the number of clusters was appointed by a cluster validation criterion, which determined the range of clusters number. There are many clusters validation criteria in the literature that could be used for our purpose. We have used the well studied case of the Xie-Beni validity index [48]. This criterion gave the best values in the range of 12–18 clusters. The final number was defined by MIPS Functional Catalogue [49], which was used to evaluate the clusters from the biological point of view by characterizing the functional distribution of every cluster with the site-defined hypergeometric distribution (p-value). This functional catalogue is organized in a hierarchical tree-like structure and consists of 28 main categories (or branches) that cover general features like cellular transport, metabolism and protein activity regulation. Proteins can be assigned to more than one functional category, allowing a multidimensional annotation scheme [50]. The best p-values (in our study we have set a maximum p-value of 10^-3 ^for accepting a functional category to characterize a cluster) were found in the case of 16 clusters.

A first observation concerning the range of the resulting cluster population is that the minimum number of genes in a cluster is 82 (i.e. in cluster 16) and the maximum is 376 (i.e. in cluster 1). In Table 2 we selectively present the functional distribution of 6 representative clusters out of the 16, from the main functional category up to the third-level subcategory. We did not take into account the rest more specific subcategories, because both the p-values and the percentage of genes were insignificant. The clusters 1, 4, 7, 16 distinguish from each other due to the fact that they contain genes with distinct biological roles, whereas clusters 3 and 6 include genes with similar biological function.

**Table 2:** MIPS functional distribution of gene representative clusters

**Cluster/Genes**	**Functional distribution (MIPS)**	**Percentage (fraction of genes)**	**p-value**
1/n = 376	14 protein fate	27.2% (102)	2.64 e-05
	14.07 protein modification	17% (64)	1.03 e-05
	11.02.03 mRNA synthesis	15.2% (57)	1.35 e-04
4/n = 259	11 transcription	29.7% (77)	6.45 e-07
	10.03 cell cycle	19.3% (50)	1.58 e-05
	10.03.01 mitotic cell cycle and cell cycle control	12.7% (33)	9.93 e-04
7/n = 189	42 biogenesis of cellular components	21.6% (41)	2.47 e-03
	14.07 protein modification	16.4% (31)	3.91 e-03
	11.01.03 mRNA processing	9% (17)	5.22 e-05
16/n = 82	16 protein with binding function or cofactor requirement	32.9% (27)	3.31 e-04
	43.01 fungal/microorganismic cell type differentiation	18.2% (15)	8.20 e-04
	43.01.03 fungal and other eukaryotic cell type differentiation	18.2% (15)	8.20 e-04
3/n = 183	01 metabolism	30% (55)	5.48 e-02
	10.03 cell cycle	14% (26)	7.48 e-02
	11.04.03 mRNA processing	8% (13)	3.57 e-03
6/n = 181	01 metabolism	39.7% (72)	3.91 e-06
	10.01 DNA processing	15.4% (28)	1.05 e-03
	11.02.03 mRNA synthesis	17.6% (32)	2.99 e-04

Examples of gene clusters after clustering the gene expression data. The first column represents the number of cluster and the number of genes in it. The second column represents the functional distribution of every cluster according to MIPS from the main category up to the third-level subcategory. The third column shows the percentage of genes belonging to this category and in the brackets the corresponding actual number. The last column displays the p-value found by MIPS in every functional category.

### Data integration

In our approach we chose to unify the above types of data for various reasons. Firstly PPI data from high-throughput techniques is currently flooded with false interactions [51]. Also protein interaction measurements descend from a certain range of experimental conditions, thus they succeed to identify only a small fraction of all possible protein-protein interactions. In addition PPI networks contain unstable interactions or interactions that take place at different time points, thus the resulting network does not represent the real one but an overlap of many different snapshots [3]. Studies that followed the direction of just clustering a PPI graph (without taking into account gene expression data) resulted in partially valid functional modules but failed in elevating those interactions that would contribute to even more coherent modules like the ones of our integrated method. There are cases where many of these algorithms tend to ignore peripheral proteins that link to protein clusters with few connections, even though many of those are true and experimentally verified interactions [3,33,42]. In our approach a lot of such interactions is not neglected because they are 'saved' by the gene expression information that is overlaid as weight on the PPI graph. However an important attribute of PPI networks is that they provide information about direct binding partners, property lost when dealing with co-expression networks. All the reasons mentioned above clarify the insufficiency of the PPI data alone to produce highly confident functional modules.

On the other side gene expression data provides information of the genome under many different experimental conditions despite the large amount of inherent noise. In literature there are studies like [36] that examined the evident modularity of co-expression networks and studies like [52] that constructed co-expression networks and indicated two genes as functionally related if their expression similarity is conserved under many different conditions and across large evolutionary distances. Also there are cases [53] where the similarity of expression profiles was used as a criterion to identify true positive interactions. Although strong expression similarity between two genes implies same transcriptional control and functional association, the yielded interactions are often indirect. In a gene co-expression network two closely connected nodes are highly correlated but this observation does not lead to direct interacting partners. Also there are cases where functionally related genes present significant difference in their expression profiles [15] (i.e. a certain gene maybe strongly suppressed in order to allow another one to be expressed). Therefore clustering them (based strictly on their expression profiles) into separate groups will lead to a loss of the specific relation. It is obvious that co-expression networks offer numerous hypotheses about the functional association among genes but cannot serve on their own as the basis for detecting functional modules. Besides the majority of contemporary methods identifies functional modules via PPI networks, where the in-between relations are distinct, and a fraction of these methods move a step forward and enrich these structures with other kinds of data [3].

In order to keep the advantages from both sides we focused on a highly confident PPI network and used the gene expression data as reinforcement. In other words the PPI network is the protagonist, whereas the gene expression data reassures the entrance of certain interactions into the modules, even if they are not favoured by the topology. In this way our goal, i.e. to identify functional modules on the PPI network, is accomplished with great success, since we manage to lessen up to a great degree the drawbacks of each kind of data.

### Weighted graph

The protein-protein interaction network of yeast is represented as a graph G(V, E). The vertices of the graph are the set of unique proteins and therefore in our case is |V| = 2985, whereas the edges of the graph represent the interactions |E| = 8081. Below we explain our methodology of adding weights to the edges by exploiting the information of the gene expression profiles mentioned above. In order to add weight to an interaction between two proteins x and y, we find the clusters C(x) and C(y) where they belong and the corresponding centroids K_x_, K_y _of these clusters. Then, we calculate the distance of each gene from its centroid and the distance between the two centroids.

The weight of the PPI interaction is given by the metric described as:

*W*(*x*, *y*) = *n*_1_(||*x *- *K*_*x*_||^2 ^+ ||*y *- *K*_*y*_||^2^) + *n*_2_||*K*_*x *_- *K*_*y*_||^2^

||·|| stands for the distance metric and there are many ways of measuring it (e.g. Euclidean). The constants n_1 _and n_2 _add an extra confidence score to the factors of the weight function. They can have the same or different values according to which member (if any) of the function we want to enforce. We have selected n_2 _> n_1 _because we consider the distance between centroids more significant comparing to the distance of each gene from its centroid. This selection was driven by the fact that there is noise (outliers) in the gene expression profiles.

Following the principle that similar expression profiles are associated with similar function [14], the value of the above stated metric favours interactions whose corresponding genes have similar expression (i.e. small weight values derive from small distance among profiles) and thus enhances them in the overall network.

On the other hand there are many cases where proteins of the same complex may have quite different (e.g. transient complexes) or even inverse expression profiles. As a result an interaction in such a pair of proteins would fail to be emphasized by the above metric and thus not preferred in comparison to another smaller weighted interaction. Nevertheless, the proposed algorithm that we will describe in detail in the following section, manages to overcome problems like these by incorporating information given by the rest weighted neighbours of such an interaction, during the construction of a functional module.

### Algorithm DMSP

In this section we will give, after some preliminary concepts, a description of the proposed algorithm named Detect Module from Seed Protein (DMSP). DMSP builds functional modules by expanding the kernel neighbourhood generated by a 'seed' protein. In the implementation presented here, both the 'seed' protein as well as the extracted functional module, are part of a larger network which can be represented by a weighted graph structure.

#### Preliminaries

As we have already mentioned, in the approach we have followed, we combine gene expression profiles and PPI data, in the form of a weighted graph, G(V, E). By N(x) we denote the neighbours of a node x, or in other words the set of nodes that are connected to x. Then, the degree of x is equivalent to the number of neighbours of x |N(x)|. For a given subgraph G_1 _of a larger graph G we define the internal degree |N_G1_^INT^| as the number of edges connecting x with other vertices belonging to G_1 _and external degree as the number of nodes with which x is connected and exist in G but do not belong to G_1_.

The above concepts can be expanded to the weighted graphs easily. Weighted degree of a node is the sum of weights of the edges between x and its neighbours divided by |N(x)|. Weighted internal degree of a node x is the sum of weights of the edges between x and its neighbours within G_1 _over |N_G1_^INT^|:

βG1INT(x)=1|NG1INT|∑y∈NG1INTwxy
 MathType@MTEF@5@5@+=feaafiart1ev1aaatCvAUfKttLearuWrP9MDH5MBPbIqV92AaeXatLxBI9gBaebbnrfifHhDYfgasaacH8akY=wiFfYdH8Gipec8Eeeu0xXdbba9frFj0=OqFfea0dXdd9vqai=hGuQ8kuc9pgc9s8qqaq=dirpe0xb9q8qiLsFr0=vr0=vr0dc8meaabaqaciaacaGaaeqabaqabeGadaaakeaaiiGacqWFYoGydaqhaaWcbaGaem4raC0aaSbaaWqaaiabigdaXaqabaaaleaacqWGjbqscqWGobGtcqWGubavaaGcdaqadaqaaiabdIha4bGaayjkaiaawMcaaiabg2da9maalaaabaGaeGymaedabaWaaqWaaeaacqWGobGtdaqhaaWcbaGaem4raC0aaSbaaWqaaiabigdaXaqabaaaleaacqWGjbqscqWGobGtcqWGubavaaaakiaawEa7caGLiWoaaaWaaabuaeaacqWG3bWDdaWgaaWcbaGaemiEaGNaemyEaKhabeaaaeaacqWG5bqEcqGHiiIZcqWGobGtdaqhaaadbaGaem4raC0aaSbaaeaacqaIXaqmaeqaaaqaaiabdMeajjabd6eaojabdsfaubaaaSqab0GaeyyeIuoaaaa@5416@

Correspondingly we define the term of weighted external degree.

The density of a graph G(V, E) is generally measured by the proportion of the number of edges in the graph to the number of all possible edges, which is equal to |V|(|V|-1) for an undirected graph. Weighted density of a graph or subgraph D_w_(G), is the sum of the weights of actual edges over the edges among all nodes in G:

Dw(G)=∑〈x,y〉∈Ewxy|V|(|V|−1)
 MathType@MTEF@5@5@+=feaafiart1ev1aaatCvAUfKttLearuWrP9MDH5MBPbIqV92AaeXatLxBI9gBaebbnrfifHhDYfgasaacH8akY=wiFfYdH8Gipec8Eeeu0xXdbba9frFj0=OqFfea0dXdd9vqai=hGuQ8kuc9pgc9s8qqaq=dirpe0xb9q8qiLsFr0=vr0=vr0dc8meaabaqaciaacaGaaeqabaqabeGadaaakeaacqWGebardaWgaaWcbaGaem4DaChabeaakiabcIcaOiabdEeahjabcMcaPiabg2da9maalaaabaWaaabeaeaacqWG3bWDdaWgaaWcbaGaemiEaGNaemyEaKhabeaaaeaadaaadaqaaiabdIha4jabcYcaSiabdMha5bGaayzkJiaawQYiaiabgIGiolabdweafbqab0GaeyyeIuoaaOqaamaaemaabaGaemOvayfacaGLhWUaayjcSdWaaeWaaeaadaabdaqaaiabdAfawbGaay5bSlaawIa7aiabgkHiTiabigdaXaGaayjkaiaawMcaaaaaaaa@4E11@

#### Description

The algorithm proposed in this paper operates in two phases. Firstly accepts one 'seed' protein and selects a subset of its most promising neighbours, subsequently expands this initial kernel to accept more proteins. This expansion is based on certain assumptions, concerning the number of neighbours for the specific protein as well as the weights of these connections.

In the first stage of the algorithm only a certain number of the neighbours of the 'seed' protein (named hereafter s) is selected. These adjacent nodes are sorted in descending degree of significance and this subset of nodes – proteins is named kernel.

The two criteria by which the original kernel is selected are the density of the kernel and the weighted internal and external degrees of it.

Initially, the kernel is equal to all the neighbours of s:

*Kernel*(*s*) = *K*_*s *_≡ *N*(*s*)

Then for each one of the neighbours u_i _belonging to Kernel(s) we find the N^INT^(K_s_), N^EXT^(K_s_), as well as the *β*^INT ^and *β*^EXT^. The objective for selecting the kernel of the seed node is two-fold. Firstly we check so that the number of edges of a kernel node within the rest of the kernel is larger or at least equal to the number of the edges that a node has outside the group. We accomplish this by requesting for the internal and external degrees of each node:

IO(Ks,ui)=|NKsINT(ui)||NKsEXT(ui)|+|NKsINT(ui)|>p1
 MathType@MTEF@5@5@+=feaafiart1ev1aaatCvAUfKttLearuWrP9MDH5MBPbIqV92AaeXatLxBI9gBaebbnrfifHhDYfgasaacH8akY=wiFfYdH8Gipec8Eeeu0xXdbba9frFj0=OqFfea0dXdd9vqai=hGuQ8kuc9pgc9s8qqaq=dirpe0xb9q8qiLsFr0=vr0=vr0dc8meaabaqaciaacaGaaeqabaqabeGadaaakeaacqWGjbqscqWGpbWtdaqadaqaaiabdUealnaaBaaaleaacqWGZbWCaeqaaOGaeiilaWIaemyDau3aaSbaaSqaaiabdMgaPbqabaaakiaawIcacaGLPaaacqGH9aqpdaWcaaqaamaaemaabaGaemOta40aa0baaSqaaiabdUealnaaBaaameaacqWGZbWCaeqaaaWcbaGaemysaKKaemOta4KaemivaqfaaOWaaeWaaeaacqWG1bqDdaWgaaWcbaGaemyAaKgabeaaaOGaayjkaiaawMcaaaGaay5bSlaawIa7aaqaamaaemaabaGaemOta40aa0baaSqaaiabdUealnaaBaaameaacqWGZbWCaeqaaaWcbaGaemyrauKaemiwaGLaemivaqfaaOWaaeWaaeaacqWG1bqDdaWgaaWcbaGaemyAaKgabeaaaOGaayjkaiaawMcaaaGaay5bSlaawIa7aiabgUcaRmaaemaabaGaemOta40aa0baaSqaaiabdUealnaaBaaameaacqWGZbWCaeqaaaWcbaGaemysaKKaemOta4KaemivaqfaaOWaaeWaaeaacqWG1bqDdaWgaaWcbaGaemyAaKgabeaaaOGaayjkaiaawMcaaaGaay5bSlaawIa7aaaacqGH+aGpcqWGWbaCdaWgaaWcbaGaeGymaedabeaaaaa@6A76@

In this study we selected p_1 _to have value over 45%. At the same time and after we have confirmed that a selected node fulfils the first condition, we request that the same node has smaller weighted internal degree than its corresponding weighted external degree:

βKsINT(x)<βKsEXT(x)
 MathType@MTEF@5@5@+=feaafiart1ev1aaatCvAUfKttLearuWrP9MDH5MBPbIqV92AaeXatLxBI9gBaebbnrfifHhDYfgasaacH8akY=wiFfYdH8Gipec8Eeeu0xXdbba9frFj0=OqFfea0dXdd9vqai=hGuQ8kuc9pgc9s8qqaq=dirpe0xb9q8qiLsFr0=vr0=vr0dc8meaabaqaciaacaGaaeqabaqabeGadaaakeaaiiGacqWFYoGydaqhaaWcbaGaem4saS0aaSbaaWqaaiabdohaZbqabaaaleaacqWGjbqscqWGobGtcqWGubavaaGcdaqadaqaaiabdIha4bGaayjkaiaawMcaaiabgYda8iab=j7aInaaDaaaleaacqWGlbWsdaWgaaadbaGaem4CamhabeaaaSqaaiabdweafjabdIfayjabdsfaubaakmaabmaabaGaemiEaGhacaGLOaGaayzkaaaaaa@43E0@

Nodes that fail to pass the above criteria are discarded, while those that do, are sorted based on the level that each one of them manages to do so.

This original subset of proteins can be furthered distilled, in order to acquire an even more coherent kernel. This can be achieved by minimizing D_w_(K_s_) as:

Dwmin⁡(Ks)=min⁡(arg⁡Ks)Dw(Ks)
 MathType@MTEF@5@5@+=feaafiart1ev1aaatCvAUfKttLearuWrP9MDH5MBPbIqV92AaeXatLxBI9gBaebbnrfifHhDYfgasaacH8akY=wiFfYdH8Gipec8Eeeu0xXdbba9frFj0=OqFfea0dXdd9vqai=hGuQ8kuc9pgc9s8qqaq=dirpe0xb9q8qiLsFr0=vr0=vr0dc8meaabaqaciaacaGaaeqabaqabeGadaaakeaacqWGebardaqhaaWcbaGaem4DaChabaGagiyBa0MaeiyAaKMaeiOBa4gaaOGaeiikaGIaem4saS0aaSbaaSqaaiabdohaZbqabaGccqGGPaqkcqGH9aqpcyGGTbqBcqGGPbqAcqGGUbGBcqGGOaakdaWfqaqaaiGbcggaHjabckhaYjabcEgaNbWcbaGaem4saS0aaSbaaWqaaiabdohaZbqabaaaleqaaOGaeiykaKIaemiraq0aaSbaaSqaaiabdEha3bqabaGcdaqadaqaaiabdUealnaaBaaaleaacqWGZbWCaeqaaaGccaGLOaGaayzkaaaaaa@4CFF@

In this step, DMSP removes one at a time, each one of the sorted per significance nodes starting from the most insignificant until it reaches a minimum value of weighted density.

The second stage of the algorithm receives as input the selected kernel of the 'seed' protein and iteratively adds adjacent nodes based again on certain criteria. The first criterion the algorithm checks is the same as the first one of the initial stage of the algorithm. After this criterion has been checked then we select a node to be added to the neighbourhood, if it satisfies the following:

Wvui≤p2⋅βGINT(v)
 MathType@MTEF@5@5@+=feaafiart1ev1aaatCvAUfKttLearuWrP9MDH5MBPbIqV92AaeXatLxBI9gBaebbnrfifHhDYfgasaacH8akY=wiFfYdH8Gipec8Eeeu0xXdbba9frFj0=OqFfea0dXdd9vqai=hGuQ8kuc9pgc9s8qqaq=dirpe0xb9q8qiLsFr0=vr0=vr0dc8meaabaqaciaacaGaaeqabaqabeGadaaakeaacqWGxbWvdaWgaaWcbaGaemODayNaemyDau3aaSbaaWqaaiabdMgaPbqabaaaleqaaOGaeyizImQaemiCaa3aaSbaaSqaaiabikdaYaqabaGccqGHflY1iiGacqWFYoGydaqhaaWcbaGaem4raCeabaGaemysaKKaemOta4KaemivaqfaaOWaaeWaaeaacqWG2bGDaiaawIcacaGLPaaaaaa@4289@

G is the final module that is built from the initial kernel (i.e. initially G = K_s_), we select the constant p_2 _to be anywhere between 0.9 and 1.0. Relation (8) states that in order for an adjacent node u_i _of some kernel node v, to become member of the module, its weight must be less or equal to a specific percentage of the weighted internal degree of node v. Below we describe the pseudocode for implementing the function of the second stage of DMSP, named Determine_Module, which is responsible for the final determination of the functional module:

Algorithm **Determine_Module**

Input: Ks, p1, p2

Output: Final Module G

   I. *G *≡ *G *∪ *K*_*s*_

   II. **For **all v G

      a. Calculate N(v)

      b. *N*(*v*) ≡ *N*(*v*) ∩ *K*_*s*_

      c. **For **every u_i _in N(v)

         i. Calculate N_G_^INT^, N_G_^EXT^

         ii. **If ***v *∈ *K*_*s*_

            1. R1: IO(G, u_i_) > p_1_

         iii. **Else**

            1. R1: IO(G, u_i_) > p_2_

         iv. **End**

         v. **if **(8) is true **then **R2 = true **else **R2 = false

         vi. **if **R1 = true **AND **R2 = true

            1. *G *≡ *G *∪ *ν*

            2. G = Determine_Module(K_s_, p_1_, p_2_, G)

         vii. **End**

      d. **End**

   III. **End**

As we can depict from lines (ii.1), (iii.1) of the pseudocode there are two percentage values describing the relation of internal and external neighbours as it is calculated in equation 5. The distinction of this value depends on whether the current node is a direct neighbour of the kernel or not. In this way we have a two-layer scheme where we retain a looser criterion for immediate neighbours and a stricter one for the remote neighbours of the initial kernel.

## Authors' contributions

IAM conceived the integration method, the algorithm, and prepared the data sets. KD was responsible for writing the main body of the text and especially for the biological aspects of the study. All the above actions were supervised by AB. All authors read and approved the final manuscript.
